# Estimation of Absolute Cardiovascular Risk in Individuals with Diabetes Mellitus: Rationale and Approaches

**DOI:** 10.5402/2011/242656

**Published:** 2011-11-23

**Authors:** Justin B. Echouffo-Tcheugui, Modele O. Ogunniyi, André P. Kengne

**Affiliations:** ^1^Department of Global Health, Rollins School of Public Health, Emory University, 1518 Clifton Road NE, Atlanta, GA 30322, USA; ^2^Division of Cardiology, Emory University School of Medicine, 49 Jesse Hill Jr. Drive SE, Atlanta, GA 30303, USA; ^3^South African Medical Research Council and University of Cape Town, P.O. Box 19070, Tygerberg, Cape Town 7505, South Africa; ^4^Julius Centre for Primary Care, University Medical Centre, Utrecht, Postbus 85500, 3508 GA Utrecht, The Netherlands; ^5^The George Institute for Global Health, The University of Sydney, P.O. Box M201, Sydney, Australia

## Abstract

*Purpose*. To examine the usefulness of cardiovascular risk estimation models in people with diabetes. *Methods*. Review of published studies that compare the discriminative power of major cardiovascular risk factors single or in combination in individuals with and without diabetes, for major cardiovascular outcomes. *Results*. In individuals with and without diabetes, major risk factors affect cardiovascular risk similarly, with no evidence of any significant interaction. Accounting for diabetes-specific parameters, cardiopreventative therapies can significantly improve risk estimation in diabetes. General and diabetes-specific cardiovascular risk models have a useful discriminative power, but tend to overestimate risk in individuals with diabetes. Their impact on care delivery, adherence to therapies, and patients' outcome remain poorly understood. *Conclusions*. The high-risk status conferred by diabetes does not preclude the estimation of absolute cardiovascular risk estimation using global risk tools in individuals with diabetes, as this is useful for the initiation and intensification of preventive measures.

## 1. Introduction

The incidence of cardiovascular disease (CVD) risk factors is higher in diabetic patients than in nondiabetic individuals [1]. Therefore, estimating global absolute cardiovascular risk in this population is essential for better risk factor management. 

Decision making and patient education in modern medicine increasingly relies on risk estimation models. A risk model is a mathematical equation that can be used to estimate the probability of having prevalent disease, incident disease, or a poor outcome among diseased individuals [2, 3]. The use of prediction models in cardiovascular medicine has become popular during the last three decades, largely driven by the Framingham study. This cohort study allowed the development of a several cardiovascular disease risk models [4–6], which have been reformulated, simplified through scores (obtained by the assignment of points to each risk factor included into the model) or charts, recalibrated to other populations, or replaced by similar models in other settings or contexts. The risk models generally estimate the probability of future events within a given time period (usually over a 5- or 10-year time horizon) in individuals with no prior cardiovascular event. This estimate depends on the combination and levels of all risk factors rather than on the presence of any single risk factor. The risk estimates are used to guide primary prevention. Modern guidelines increasingly emphasize the importance of estimating the individual's global cardiovascular risk as a more appropriate basis for risk factor management. 

Three different approaches have been used to account for exposure to chronic hyperglycemia in CVD risk estimation. The first approach includes diabetes status in the risk tool such that, all things being equal, a diabetic patient will always have a higher risk than a nondiabetic patient with a similar risk factor profile such as blood pressure. The second approach builds separate models for individuals with and without diabetes. In this approach, the knowledge of risk factor effect in the diabetic population does not inform CVD risk estimation in the nondiabetic population. Finally, the third approach classifies individuals with diabetes as having a risk level equivalent to that of individuals without diabetes who survived a cardiovascular event, making them eligible for preventive therapies without further CVD risk estimation. However, this does not account for the fact that CVD risk is not uniformly distributed, but follows a gradient from the lowest to the highest risk [7]. 

In this paper, we examine the utility of existing CVD risk estimation models (also referred to as absolute risk equations) in individuals with diabetes. We attempt to answer three key questions. (1) How does CVD risk factor profile differ among individuals with diabetes and nondiabetic individuals? (2) What are the opportunities for refining CVD risk estimation afforded by the chronic dysglycemic environment? (3) Does the performance of CVD risk estimation tools developed specifically for individuals with diabetes differ from tools developed for the general population? 

## 2. CVD Risk Factor Profile among Individuals with Diabetes and Nondiabetic Individuals

### 2.1. Blood Pressure and CVD Risk among Individuals with Diabetes and Those without Diabetes

Blood pressure (BP) is a major determinant of cardiovascular risk. Compared to individuals without diabetes, average BP levels in individuals with diabetes are generally higher. Consequently, a categorization of the population based on arbitrary BP cutoff levels will amount to a higher prevalence of hypertension among individuals with diabetes than in the rest of the population [8–10]. On this basis, it has generally been known that there is a higher cardiovascular risk related to BP among individuals with diabetes than nondiabetic individuals. Such an approach is simplistic, especially given the continuous association between BP and CVD risk without evidence of a threshold below which the risk is stable or null. At each BP level, both the absolute and relative CVD risk levels may be higher among individuals with diabetes. Therefore, a key question is whether any change in BP level confers the same level of relative risk among individuals with diabetes and those without diabetes. In other words, is there an interaction between diabetes and BP to affect CVD risk? 

This question has been examined in many studies over the last four decades. At least three studies concluded an interaction, with a higher risk among individuals with diabetes in one study [11] or in those without diabetes in two studies [12, 13]. A much larger, better-designed, and well-conducted study, the APCSC meta-analysis, did not find an interaction between diabetes and blood pressure [14]. In this study, the association between systolic BP and the risk of multiple cardiovascular events was linear and continuous (on the log scale) in individuals with and without diabetes [14]. Each 10 mm Hg increase in SBP was associated with the same relative risk of cardiovascular event among individuals with diabetes and those without diabetes [14]. 

### 2.2. Lipid Levels and Risk of Cardiovascular Diseases among Individuals with Diabetes and Those without Diabetes

It is generally known that the “diabetes dyslipidemia” is typically characterized by high levels of triglycerides, low levels of HDL cholesterol, and normal or near-normal levels of LDL cholesterol [15]. This description has fueled a debate around two main questions. (1) Which lipid variable is most correlated with CVD risk among individuals with diabetes? (2) Do all lipid variables confer the same level of CVD risk in individuals with diabetes and those without the condition? 

We will focus on the second question. A few studies have directly compared the relative effect of various lipid variables on CVD risk among individuals with diabetes and those without diabetes. Regarding total and LDL cholesterol, the Multiple Risk Factors Intervention Trial (MRFIT) suggested that total cholesterol is associated with a lower relative CVD risk among individuals with diabetes compared to those with the disease [13]. However, the vast majority of studies, including the APCSC study, found no interaction between diabetes, total and HDL cholesterol, and CVD risk [14]. As previously observed, the APCSC study showed that triglycerides, HDL and non-HDL-cholesterol, and total/HDL cholesterol ratio influence CVD risk similarly among individuals with diabetes and those without diabetes. 

### 2.3. Smoking and Cardiovascular Risk among Individuals with Diabetes and Those without Diabetes

Existing studies concur on the fact that prevalence of smoking among individuals with diabetes is not appreciably different from that in the general population [16, 17]. One study has indicated that smoking could account for up to 65% of cardiovascular deaths in people with diabetes; however, studies that have investigated the statistical interaction between diabetes and smoking on the risk of major cardiovascular outcomes have generated conflicting results [1, 13, 18–22]. Nevertheless, the trends indicated an absence of interaction effect between smoking and diabetes on the CVD risk in most studies including Framingham study [1], the Paris Prospective study [20], and the APCSC study [23].

## 3. Refinement of CVD Risk Estimation among Individuals with Diabetes—Opportunities and Challenges

The aforementioned findings indicating that traditional risk factors used for CVD risk estimation affect CVD risk among individuals with diabetes and those without diabetes, similarly, argue against a development of separate risk models for these two groups. However, given the specific distribution of some of risk factors in the context of diabetes, these can contribute to an improvement or a modification of risk prediction among individuals with diabetes, but not necessarily among those without diabetes. This is particularly the case for diabetes-specific CVD risk factors and preventive therapies. 

### 3.1. Cardiovascular Risk Factors Specific to Diabetes

Markers of chronic hyperglycemia conventionally used for the diagnosis and control of diabetes are independent risk factors for CVD. Indeed, fasting and postprandial glucose levels, as well as hemoglobin A1c (HbA_1c_) are linearly associated with CVD risk among individuals with and without diabetes [24, 25]. The inclusion of markers of chronic hyperglycemia such as HbA_1c_ used as a continuous variable in risk models does not necessarily improve CVD risk estimation in the nondiabetic population [26]. Inclusion of diabetes status as a binary variable further decreases the extent of the contribution of glycemia to CVD risk prediction among individuals without diabetes, thus making it less useful to CVD risk discrimination. This is not the case for those with diabetes, among whom the contribution of these parameters has been unequivocally demonstrated [27, 28]. 

Some microvascular complications of diabetes are independent CVD risk markers and are thus informative and contribute to prediction even in the presence of other known risk factors. Microvascular complications that have been used in CVD risk estimation tools include diabetic retinopathy and microalbuminuria, derived from risk prediction models employed in the ADVANCE study [27]. 

Studies have suggested that age and diabetes interact to affect CVD risk [29], with age having a multiplicative effect after the diagnosis of diabetes, thus justifying its inclusion in diabetes-specific tools as two parameters: age at diagnosis of diabetes and duration of diabetes [29]. This consideration has largely influenced the development of the United Kingdom Prospective Diabetes Study (UKPDS) risk scores [29, 30], that has been used in more recent models [27, 31, 32]. 

### 3.2. Preventive Cardiovascular Therapies

Contemporary management algorithms assume that patients in general and especially individuals with diabetes are naive of preventive treatment, at least until the diagnosis of diabetes. However, in practice, the vast majority of individuals with diabetes receive at least one cardiopreventive medication, generally blood pressure or lipid-lowering medications, or aspirin [33]. These medications are used less frequently in the general population. Given that medications modify the expression of risk factors, they should be accounted for in the estimation of global CVD risk. Very few models have actually accounted for the effect of treatment [34]. Some risk models have attempted to do this by including blood pressure treatment in CVD risk models [6]. This approach however is not optimal, as it does not account for the fact that medications are more likely to be prescribed to individuals with diabetes than those without diabetes. Hence, the effect of treatment for high blood pressure in a mixed population (individuals with and without diabetes) will be diluted by the lower levels of prescription among those without diabetes. This indicates the need for more appropriate models of CVD risk estimation among individuals with diabetes, especially among those already receiving several preventative therapies. 

## 4. Performance of Cardiovascular Risk Estimation Tools among Individuals with Diabetes

The number of CVD risk models developed in the general population or among individuals with diabetes has been increasing. A systematic review of these tools is beyond the scope of this paper [27, 28]. However, the vast majority of available models was developed in predominantly nondiabetic populations and has hardly been validated in exclusively diabetic populations. 

Generally, risk models have been developed using logistic regressions or survival analyses. The coefficients of different factors included in the final equation indicate their relative contribution to the overall CVD risk [2]. The validation of a model consist of testing whether the risk tool correctly estimates the probability of future events in one or several populations other than the one in which it was developed. The two characteristics that are generally evaluated are discrimination and calibration [2]. Discrimination is the ability to correctly classify individuals who go on to develop a cardiovascular event and those who remain event free [2]. For example, if there are two individuals with diabetes, with one developing a cardiovascular event after certain time and the other remaining CVD free, a model with a high discriminative ability will systematically assign a higher risk to the first subject compared to the second. Calibration is the extent to which predicted risk from a model matches observed risk in the population [2]. For instance, a 5-year estimated probability of cardiovascular disease of 20% for a patient means that, in a given group of patients with similar characteristics, 20% will experience a cardiovascular event within a 5-year period. 

Framingham CVD risk equations for the general population [33, 35–38] and diabetes-specific CVD risk tools such as those derived from the UKPDS study [33, 37–39] have been tested independently among individuals with diabetes. These models generally include age or its component, sex, blood-pressure-related variables, levels of various blood lipid variables, and smoking. Available studies suggest that the discriminative power of these CVD risk equations among individuals with diabetes varies for low to acceptable for CVD risk estimation. Recent studies have also indicated a tendency toward systematic overestimation of CVD risk by the two groups (Framingham and UKPDS) of equations among individuals with diabetes [27, 33]. Some of the studies that compared these equations did not find a major difference [37, 40–42]; others found a better estimation with the used of the UKPDS tools [33, 38]. These studies have shown that the absolute CVD risk is not uniformly distributed among individuals with diabetes [7]. It follows a gradient from low to high risk, thus adequately capturing this gradient in risk estimation may allow improvement of therapy and intensification of prevention among individuals with diabetes [7]. 

## 5. Effect of the Use of CVD Risk Tools on Provider and Patient Behaviors

Whether the adoption of CVD risk models is accompanied by changes in healthcare provider and patient behaviors and thus a better adherence with preventive cardiovascular measures has not been extensively explored. To our knowledge, no study has addressed this question exclusively among individuals with diabetes. In the US-based Atherosclerosis Assessment Via Total Risk trial (AVIATOR) [43], which included 368 participants from the general population, free of prior CVD and not on statin therapy, who were randomly assigned to intervention and control arms. In the intervention group, the 10-year absolute CVD risk was computed using the Framingham equation. The risk estimation was then translated in simple terms and appended to the medical file of each patient. In the control group, preventive measures indicated in the existing guidelines were appended to the medical file of patients. During followup, the level of statin prescription was similar among high-risk patients (10-year risk > 20%) in both groups. However, in the control group, more participants with moderate risk (10–19%)—and thus eligible for statin prescription according to existing guidelines—received statins compared to the corresponding intervention arm. Among the low-risk group (<10%), there was a significant higher level of statin prescription in the intervention group. Physicians in the intervention group tended to recommend smoking cessation and those in the control group to recommend dietary changes [43]. More recently, a UK-based study examined the effect of CVD risk communication on patients' health-related behaviors. This randomized trial allocated 194 adults (with and without diabetes) with a CVD risk ≥20% to two groups, one receiving personalized information on their level of risk and the other no information on their risk level. There was a slight, although nonsignificant, increase in objectively measured physical activity levels (using accelerometers) over a one-month period [44]. These results indicate the need to rethink risk communication strategies to patients in order to influence their behaviors. 

## 6. Conclusions

CVD remains the leading cause of disability and death among individuals with diabetes. It is therefore important for individuals with diabetes to have access to more precise and useful information on their CVD risk level, in order to modify their health-related behaviors. Healthcare providers also need instruments that will help in educating diabetic patients on their risk of major events (e.g., myocardial infarction and stroke) and to initiate appropriate preventive interventions (pharmacological and nonpharmacological). Compared to nondiabetic subjects, individuals with diabetes generally have a higher CVD risk and deserve due attention. However, the CVD risk is not uniformly distributed in individuals with diabetes, but rather follows a gradient [7]. Adequately capturing this gradient does not only depend on individual risk factors but on their combination, thus the need for an estimation of global CVD risk among individuals with diabetes [45]. Thus, the use of CVD risk estimation tools is probably beneficial for the prevention of cardiovascular events in patients with diabetes, especially as the performance of these tools has generally improved over time, and the inclusion of diabetes-specific risk factors has helped to refined risk estimation.
